# SF3B1-mutated MDS coexisting with β-thalassemia trait: A case report and literature review focused on diagnostic distinction from acquired β-thalassemia

**DOI:** 10.1097/MD.0000000000051047

**Published:** 2026-10-09

**Authors:** Zenghui Fang, Lu Chen, Menghe Zhao, Xin Chen

**Affiliations:** a Department of Clinical Laboratory, Affiliated Jinhua Hospital, Zhejiang University School of Medicine, Jinhua, China; b Hangzhou Hospital of Traditional Chinese Medicine, Hangzhou, China.

**Keywords:** MDS, microcytic anemia, thalassemia

## Abstract

**Rationale:**

β-Thalassemia trait and myelodysplastic syndrome (MDS) may occur in the same patient. In patients with MDS and microcytosis, an important diagnostic consideration is whether the thalassemic phenotype represents a preexisting hereditary disorder or an acquired abnormality related to the myeloid clone. We report on a patient with SF3B1-mutated MDS and β-thalassemia trait, supported by longitudinal hematologic and molecular findings, and discuss the diagnostic distinction between these conditions.

**Patient concerns:**

An 81-year-old male presented with fatigue and fever, and laboratory investigations revealed microcytic anemia. Further evaluation identified SF3B1-mutated MDS with concomitant β-thalassemia trait.

**Diagnoses:**

The patient was diagnosed with SF3B1-mutated MDS with concomitant β-thalassemia trait based on bone marrow morphology and molecular studies.

**Interventions and outcomes:**

Following conservative management, the patient was discharged. During the available follow-up period, the patient remained clinically stable across three outpatient visits for complete blood count monitoring.

**Lessons:**

This case illustrates the importance of distinguishing hereditary β-thalassemia from acquired thalassemic phenotypes in patients with MDS and microcytosis. Longitudinal hematologic history, hemoglobin analysis, and molecular testing can help determine whether microcytosis predates the clonal myeloid disorder. The available case-based literature does not permit a reliable estimate of the prevalence of this co-occurrence or comparison with a control population.

## 1. Introduction

Myelodysplastic syndrome (MDS) is a clonal myeloid neoplasm characterized by ineffective hematopoiesis leading to peripheral blood cytopenias, morphological dysplasia, and cytogenetic abnormalities. It is distinguished from acute myeloid leukemia (AML) by a blast threshold of < 20% and the absence of specific AML-defining genetic rearrangements. ^[1]^ The 2022 World Health Organization (WHO) classification and the International Consensus Classification (ICC) system categorize MDS based on specific genetic abnormalities – such as del (5q), SF3B1 mutation, and biallelic TP53 inactivation – in conjunction with morphological features.^[2]^ The ATRX (alpha-thalassemia mental retardation X-linked) gene, an X-linked member of the SWI/SNF family, functions as a transcriptional co-regulator involved in the epigenetic control of gene expression.^[3,4]^ Somatic mutations in ATRX have been reported in myeloid neoplasms including MDS and can disrupt α-globin expression, leading to an acquired form of α-thalassemia termed ATMDS.^[5–7]^ Myeloid neoplasms associated with acquired β-thalassemia (BTMDS) are considerably less frequently reported than ATMDS, with only isolated cases described in the literature.^[8–10]^ Separately, MDS or related myeloid neoplasms occurring in patients with hereditary β-thalassemia have been described in a small number of published cases.^[11–15]^ These reports do not provide a defined population denominator and therefore cannot establish the prevalence of this co-occurrence. In the present case, the principal diagnostic issue was to distinguish a preexisting hereditary β-globin disorder from an acquired thalassemic phenotype arising as part of the myeloid clone. We therefore present a patient with SF3B1-mutated MDS and β-thalassemia trait supported by longitudinal hematologic findings and HBB testing, with emphasis on the diagnostic distinction and comparison with previously reported cases.

## 2. Case presentation

An 81-year-old male presented to our outpatient clinic with complaints of fatigue and fever. His medical history included a partial gastrectomy (Billroth II), hypertension, and atrial fibrillation. He had not received iron supplementation and did not report the use of iron-containing preparations for anemia before admission. His daily medication consisted of one nifedipine tablet, and all preexisting conditions were well-controlled. An electrocardiogram, as well as abdominal and pelvic computed tomography scans, revealed no significant abnormalities. Laboratory tests revealed microcytic hypochromic anemia, with marked anisocytosis and variable poikilocytosis of the peripheral red cells. The pronounced red-cell size heterogeneity was consistent with the elevated RDW of 23.6%.

The autoimmune evaluation did not support a systemic autoimmune disease; rheumatoid factor and anti-proteinase 3/myeloperoxidase antibodies were negative, while antinuclear antibody (ANA) positivity was not accompanied by relevant clinical features or other supportive serologic findings. Serological tests for infection, such as C-reactive protein and serum amyloid A, were within normal limits. Levels of immunoglobulins (IgA, IgG, IgM), free light chains (kappa, lambda), and complement components (C3, C4) were also unremarkable. Key abnormal laboratory findings on admission are summarized in Table 1.

**Table 1:** Laboratory findings at admission.

Inspection data	Value	Unit	Normal reference range
Red blood cell	3.19↓	10^12^/L	4.3–5.8
Hemoglobin	63↓	g/L	130–175
HCT	20.6↓	%	40.0–50.0
MCV	64.4↓	fl	82.0–100.0
MCH	19.7↓	pg	27.0–34.0
MCHC	306↓	g/L	316–354
RDW	23.6↑	%	11.6–14.8
β2-MG	5.54↑	mg/L	1.09–2.53
G6PD	5168↑	U/L	1300–3600
SI	183↑	ug/dl	60–170
SF	559.22↑	ng/ml	23.90–336.20
Vitamin B12	713.76↑	pmol/L	107–675
CA19–9	56.28↑	U/ml	<35
LAM	2.59↑	g/L	0.90–2.10
ANA	Positive		Negative
HbA	94.70	%	94.5–97.5
HbF	0.30	%	0.0–2.0
HbA2	5.00↑	%	2.5–3.5

ANA = anti-nuclear antibody, HbA = Hemoglobin A, HbA2 = hemoglobin A2, HbF = Fetal hemoglobin, HCT = Hematocrit, LAM = Blood light-chain LAMBDA, MCH = Mean corpuscular hemoglobin, MCHC = Mean corpuscular hemoglobin concentration, MCV = Mean corpuscular volume, RDW = Red blood cell distribution width, SF = Serum Fertin, SI = serum iron, β2-MG = β2 microglobulin.

The elevated serum iron and ferritin levels, together with marked hemosiderin deposition in the bone marrow, argued against iron deficiency as the main cause of microcytosis and were more consistent with ineffective erythropoiesis and iron accumulation in the setting of MDS with ring sideroblasts. The increased glucose-6-phosphate dehydrogenase (G6PD) activity did not indicate G6PD deficiency. Other abnormalities, including increased β2-microglobulin, vitamin B12, CA19-9, lambda light chain, and ANA positivity, were interpreted cautiously because they were not accompanied by corresponding clinical, imaging, or serologic evidence of systemic autoimmune disease, plasma cell disorder, or solid tumor during the available evaluation. These findings were therefore described as associated laboratory abnormalities rather than as separate diagnoses.

There was no reported family history of inherited disorders. However, a review of past medical records and blood tests consistently indicated microcytic anemia, while white blood cell and platelet counts remained normal. The patient’s family confirmed that he had a long-standing history of anemia since early adulthood, which had not been previously investigated. See Table 2 for detailed historical laboratory trends.

**Table 2:** Historical hematologic findings demonstrating long-standing microcytic anemia.

Year						
	2018	2019	2020	2021	2022	2022
Red blood cell (10^12^/L)	3.94	3.44	4.09	3.93	4.03	4.62
Hemoglobin (g/L)	76	64	79	74	77	87
MCV (fl)	60.4	62.1	63.5	62.1	61.8	62.1

MCV = Mean corpuscular volume.

The bone marrow aspirate morphology demonstrated anisocytosis, microcytosis, and enlarged central pallor of mature erythrocytes. Erythroblasts appeared small with scanty, basophilic cytoplasm, irregular contours, pyknotic nuclei, and occasional internuclear bridging. Macrophages showed marked hemosiderin deposition, and ring sideroblasts accounted for 25% of erythroid precursors (Fig. 3).Genetic profiling for MDS identified an SF3B1 (c.1866G > C) mutation and a KMT2A c.3926C > T (p.P1309L) variant, as shown in Table 3A.The KMT2A variant had a variant allele frequency (VAF) of 48.61% and was classified as a Class III variant of uncertain significance (VUS). Although the VAF near 50% raised the possibility of a heterozygous germline origin, paired non-hematopoietic germline testing was not available. Therefore, the origin of this variant could not be definitively established, and it was not interpreted as an acquired MDS driver. Screening for 55 common leukemia-related fusion genes returned negative results.

**Table 3:** Molecular genetic findings in the current case: MDS-related gene variants and β-thalassemia genetic testing.

A.					
Gene/transcript number	nucleotide change	Amino acid changes	Exon location	Mutation ratio/copy number	Variation grading
SF3B1NM_012433.4	c.1866G > C	p.E622D	exon14	26.50%	I
KMT2ANM_001197104.2	c.3926C > T	p.P1309L	exon7	48.61%	III

B.					
Gene	Type of test		Mutant site		Result
α- globin gene	α mutation		NA		Normal
β- globin gene	β mutation		CDs41–42 (-CTTT)		β^0^/β

**Figure 1.**
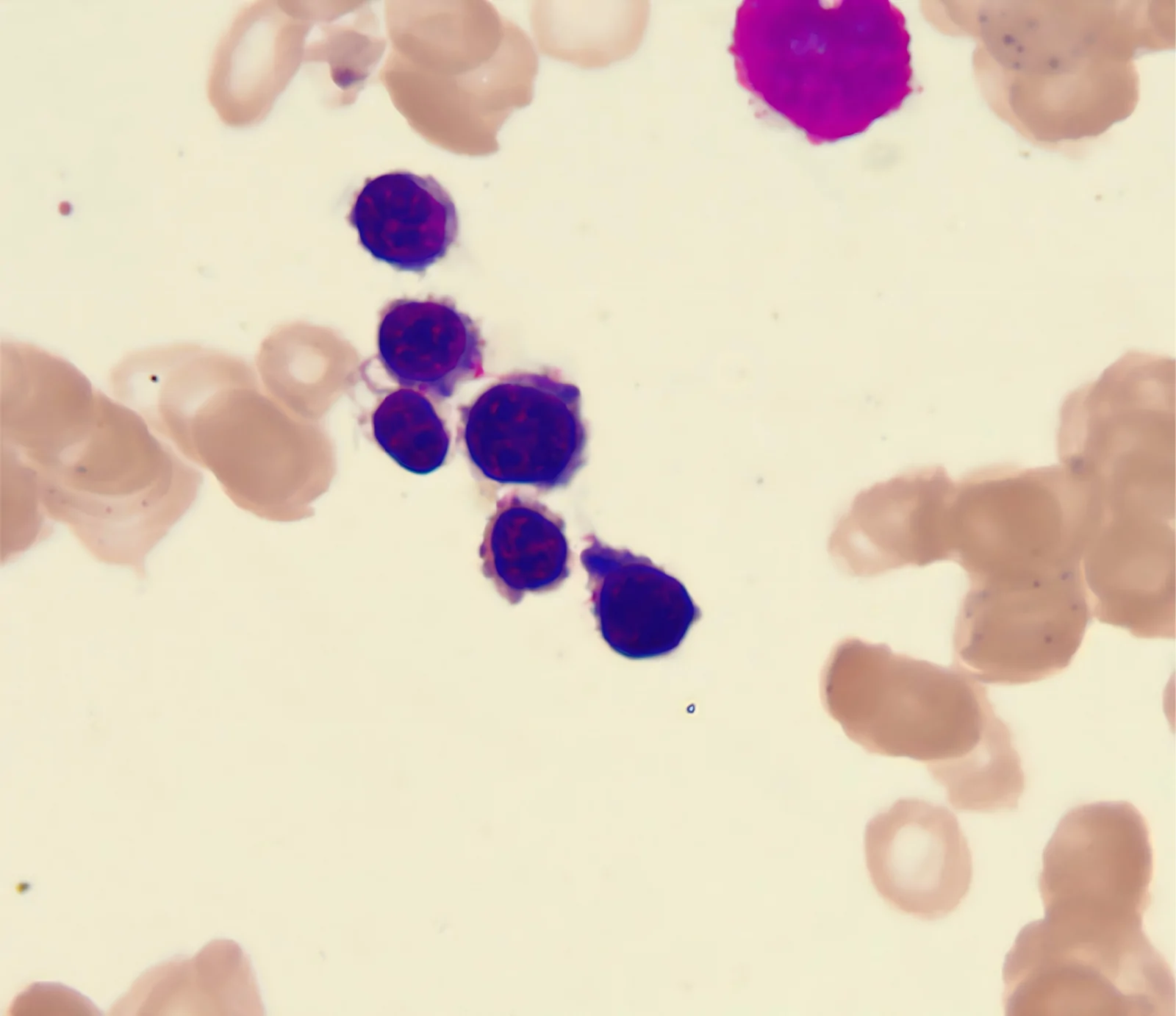
Bone marrow smear reveals significant erythroid dysplasia, characterized by microcytosis, anisocytosis, and irregular nuclear contours (Giemsa stain, 1000x magnification).

**Figure 2.**
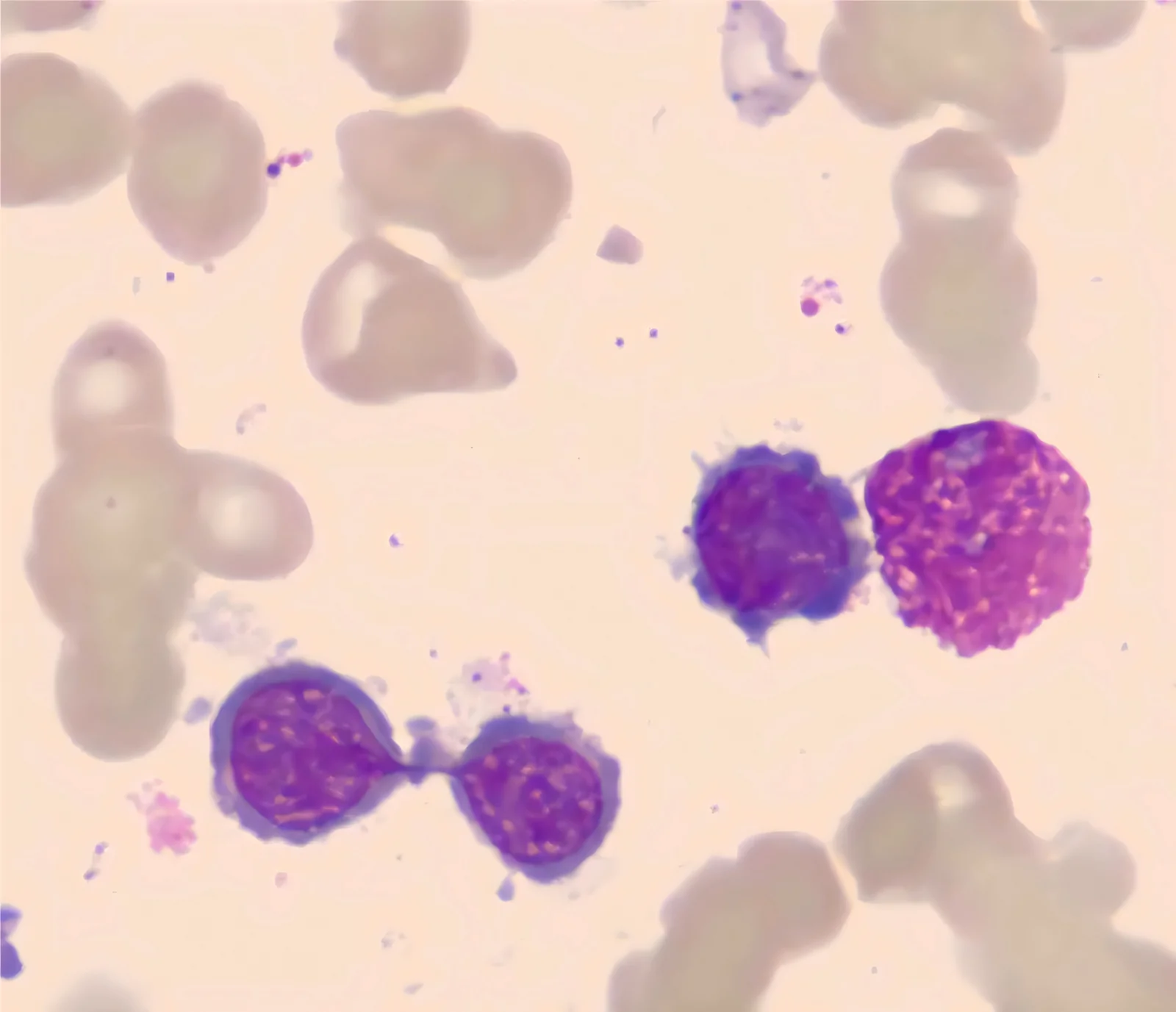
A prominent internuclear bridge is visible between two erythroblasts, a key feature of dyserythropoiesis (Giemsa stain, 1000x magnification).

**Figure 3.**
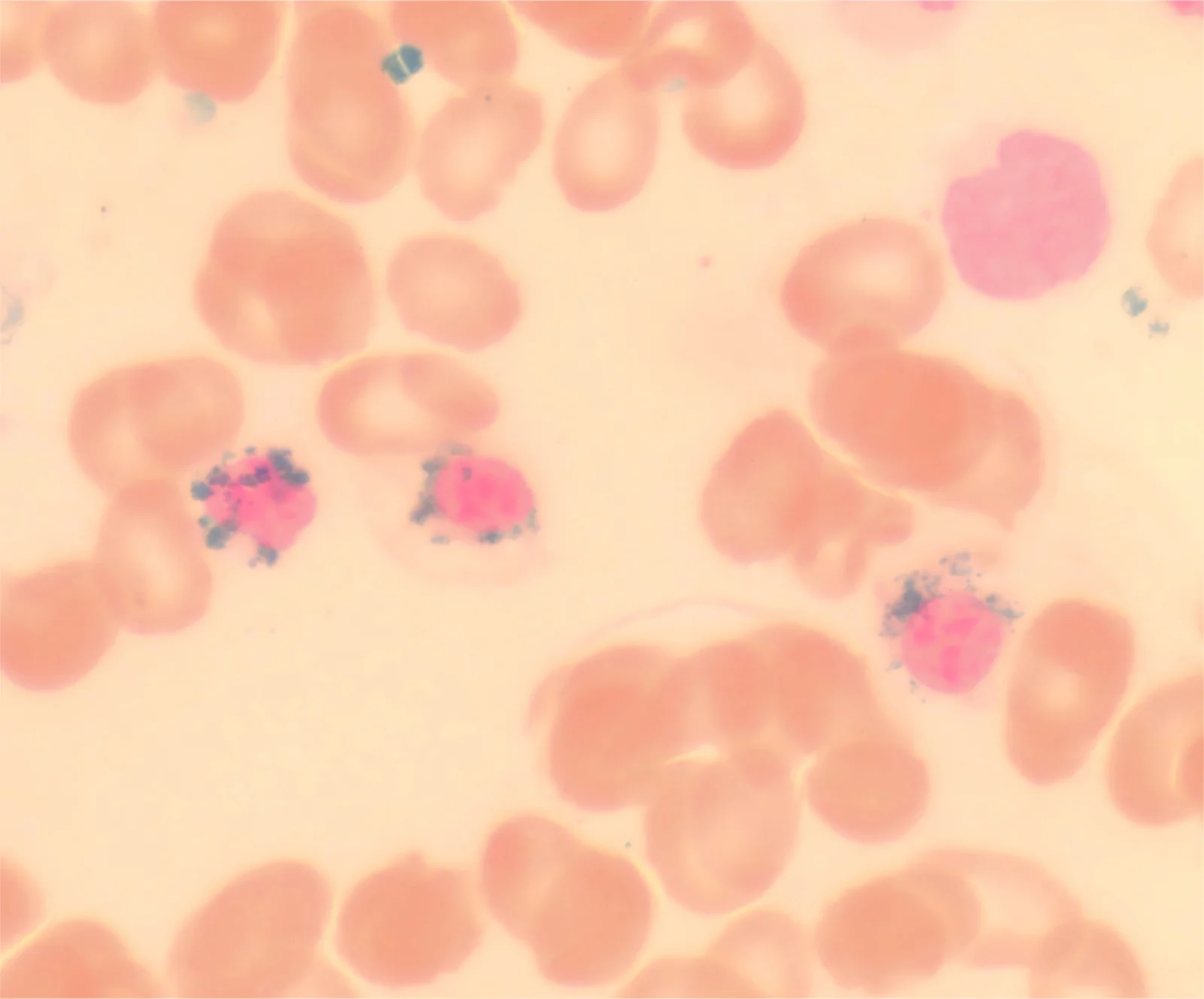
Perls’ Prussian Blue iron stain confirms the presence of numerous ring sideroblasts (arrow), which accounted for 25% of all erythroid precursors (Perls’ Prussian Blue stain, 1000x magnification).

Hemoglobin electrophoresis showed hemoglobin A (HbA) 94.70%, fetal hemoglobin (HbF) 0.30%, and hemoglobin A2 (HbA2) 5.00%, with the HbA2 level exceeding the laboratory reference range of 2.5% to 3.5%. Given the elevated HbA2 level, genetic testing for thalassemia was performed, which identified a heterozygous CD41–42 (-CTTT) mutation in the β-globin gene, consistent with β-thalassemia trait (Table 3B).Based on the collective clinical and laboratory findings, the diagnosis was SF3B1-mutated MDS with concomitant β-thalassemia trait. Because conventional cytogenetic analysis was not performed, a complete assessment of cytogenetic abnormalities relevant to WHO/ICC classification was not possible, and formal Revised International Prognostic Scoring System (IPSS-R) or Molecular International Prognostic Scoring System (IPSS-M) risk categories were not assigned.^[2,16–18]^

Due to the patient’s advanced age and family preference, he was discharged and managed conservatively. During the available short-term follow-up period, the patient completed three outpatient visits at our institution. His clinical condition and hematologic findings remained generally stable during this limited observation period, with the results summarized in Table 4.No additional long-term follow-up information was available. This case report was approved by the institutional review committee of Affiliated Jinhua Hospital, Zhejiang University School of Medicine (No. GA2025-25). Written informed consent was obtained from the patient for publication of the potentially identifiable images and clinical data included in this article.

**Table 4:** Hematologic findings during short-term outpatient follow-up.

Date			
	2023.12.11	2024.01.08	2024.02.05
Red blood cell (10^12^/L)	3.80	3.98	4.07
Hemoglobin (g/L)	78	80	82
MCV (fl)	65.1	63.5	63.5

MCV = Mean corpuscular volume.

## 3. Discussion

We report a case of SF3B1-mutated MDS co-occurring with β-thalassemia trait. The principal diagnostic issue was to distinguish a preexisting hereditary β-globin disorder from an acquired thalassemic phenotype related to the MDS clone. This distinction is clinically relevant because both conditions may contribute to ineffective erythropoiesis and microcytic anemia, but they represent different underlying disease processes.

Acquired thalassemia refers to the development of a thalassemic phenotype caused by somatic abnormalities within a hematopoietic clone, rather than by inherited germline defects of globin genes. It has been most frequently described as acquired α-thalassemia in association with MDS or other hematologic malignancies, in which impaired α-globin expression leads to globin-chain imbalance and a microcytic, hypochromic phenotype.^[19]^The best-characterized molecular mechanism is somatic alteration of ATRX, a chromatin-remodeling regulator involved in the control of α-globin gene expression.^[5]^ In contrast, acquired β-thalassemia is much rarer, and reported BTMDS cases have included acquired deletion of the β-globin gene cluster on chromosome 11, resulting in reduced β-globin production as part of the myeloid clone.^[10]^Therefore, in patients with MDS and microcytosis, acquired thalassemia should be considered in the differential diagnosis, particularly when the onset of microcytosis is new or cannot be explained by an inherited hemoglobinopathy.

Published reports of hereditary β-thalassemia coexisting with MDS or related myeloid neoplasms remain limited and heterogeneous.^[11–15]^ As summarized in Table 5, the reported cases differ in β-thalassemia phenotype, patient age, myeloid classification, ring sideroblast burden, cytogenetic findings, and accompanying molecular abnormalities. The cases reported by Ahlstedt et al and Hojat et al are the most comparable to the present patient because both involved β-thalassemia trait with SF3B1-mutated MDS and ring sideroblasts.^[12,13]^ Chen et al reported an additional inherited α-globin gene triplication, whereas Trajkova et al described MDS with possible del (5q).^[14,15]^ This heterogeneity, together with the absence of a defined source-population denominator in the available reports, prevents a reliable estimate of the prevalence of co-occurrence and does not permit a meaningful control-group comparison. Therefore, the present case is interpreted as a diagnostic comparison within the existing case-based literature rather than as evidence of an epidemiologic association.

**Table 5:** Comparison of reported cases of MDS or related myeloid neoplasms associated with hereditary β-thalassemia.

Report	β-thalassemia background	Myeloid diagnosis	Key marrow or molecular findings	Main comparison with the current case
Gologan^[11]^	β-thalassemia intermedia	RARS-T, an MDS/MPN overlap neoplasm	Worsening preexisting anemia, ring sideroblasts, and thrombocytosis; molecular findings were not reported	Shared hereditary β-thalassemia and ring sideroblasts; differed in the intermedia phenotype, younger age, female sex, and thrombocytosis
Ahlstedt et al^[12]^	β-thalassemia trait; heterozygous HBB GLN39X mutation	MDS-RS	More than 15% ring sideroblasts; SF3B1 and TET2 mutations	Shared β-thalassemia trait, SF3B1 mutation, and ring sideroblasts; differed in the HBB variant, TET2 co-mutation, and younger age
Hojat et al^[13]^	β-thalassemia trait; heterozygous HBB IVS-II-654 C > T mutation	MDS-RS	More than 90% ring sideroblasts; SF3B1 p.K700E mutation; loss of chromosome Y	Shared β-thalassemia trait, SF3B1 mutation, and erythroid dysplasia; differed in the HBB variant, ring sideroblast burden, normocytic presentation, and transfusion dependence
Chen et al^[14]^	β-thalassemia with α-globin gene triplication	MDS with SF3B1 mutation	Combined inherited globin imbalance and SF3B1-mutated clonal disease	Shared hereditary β-thalassemia and SF3B1 mutation; differed by the additional α-globin gene triplication
Trajkova et al^[15]^	β-thalassemia trait supported by family history and hemoglobin electrophoresis	MDS with possible del (5q)	Erythroid dysplasia and possible del (5q); SF3B1 mutation was not reported	Shared long-standing β-thalassemia followed by worsening anemia; differed in the macrocytic shift and possible del (5q)
Current case	β-thalassemia trait; heterozygous HBB CD41-42 (-CTTT) mutation	SF3B1-mutated MDS	25% ring sideroblasts; SF3B1 c.1866G > C mutation; KMT2A c.3926C > T (p.P1309L), Class III VUS (VAF 48.61%)	Oldest patient; persistent microcytosis; no thrombocytosis; stable only during the available short-term follow-up

Multiple lines of clinical and laboratory evidence in this case favored a preexisting hereditary β-thalassemia trait rather than acquired BTMDS. The patient had a long-standing history of anemia since early adulthood, and previous medical records consistently showed microcytic anemia with relatively stable white blood cell and platelet counts (Table 2). This clinical history substantially predated the likely onset of MDS and therefore favored a preexisting hereditary disorder. In addition, hemoglobin electrophoresis showed HbA 94.70%, HbF 0.30%, and an elevated HbA2 level of 5.00% (reference range, 2.5–3.5%). Genetic testing further identified a heterozygous CD41–42 (-CTTT) mutation in the β-globin gene, providing molecular evidence supporting inherited β-thalassemia trait (Table 3B). Taken together, these findings strongly supported a preexisting hereditary condition, although the absence of chromosome-level testing precluded absolute exclusion of a rare acquired structural event.

The diagnosis of MDS was supported by both morphological and molecular findings. The presence of 25% ring sideroblasts in the bone marrow was an important morphological feature and was consistent with the identified SF3B1 (c.1866G > C) mutation.^[20,21]^ SF3B1 encodes a core component of the U2 snRNP spliceosome, and mutations in this gene can lead to aberrant splicing of transcripts involved in mitochondrial iron homeostasis, such as ABCB7, thereby contributing to ring sideroblast formation.^[22,23]^

The KMT2A c.3926C > T (p.P1309L) variant detected in this patient had a VAF of 48.61% and was classified as a Class III VUS. A VAF close to 50% is compatible with a heterozygous germline variant; however, VAF alone cannot reliably distinguish germline from somatic origin in tumor-only sequencing.^[24]^ Because paired non-hematopoietic germline testing was not performed, the origin of this variant could not be definitively established. Accordingly, we did not consider this KMT2A variant an MDS driver and did not use it for diagnostic classification or mechanistic interpretation.

The markedly elevated red cell distribution width (RDW) of 23.6% required additional consideration because β-thalassemia trait alone did not fully account for the degree of red-cell heterogeneity. The associated peripheral red-cell morphology was characterized by marked anisocytosis with variable poikilocytosis in a microcytic, hypochromic background. In this case, the pronounced anisopoikilocytosis and RDW elevation were interpreted as reflecting superimposed MDS-related dyserythropoiesis on preexisting inherited β-thalassemia trait. This interpretation was further supported by the bone marrow findings of dysplastic erythropoiesis and 25% ring sideroblasts, together with elevated serum iron and ferritin rather than biochemical evidence of iron depletion (Table 1; Figs. 1–3). Thus, the RDW abnormality was considered to reflect the coexistence of two erythroid disorders rather than β-thalassemia trait alone.

We acknowledge that conventional cytogenetic analysis was not performed in this case. This is an important limitation because chromosome-level abnormalities relevant to MDS classification and prognostic assessment were not systematically evaluated.^[2,16–18]^ In particular, del (5q), complex cytogenetic abnormalities, and other structural changes could not be fully excluded. A large deletion or structural rearrangement involving the β-globin gene cluster on chromosome 11, as previously reported in acquired β-thalassemia associated with MDS, also cannot be excluded with complete certainty.^[10]^ We therefore use the descriptive term SF3B1-mutated MDS rather than the formal subtype label MDS-SF3B1 and do not report a formal IPSS-R or IPSS-M risk category. This limitation does not negate the morphologic and molecular evidence supporting MDS, but it restricts the certainty of subtype classification and prognostic assessment. Nevertheless, the long-standing microcytic anemia since early adulthood, HbA2 level of 5.00%, and pathogenic heterozygous HBB CD41–42 (-CTTT) mutation continue to strongly support preexisting hereditary β-thalassemia trait rather than an acquired thalassemic phenotype.

The single-case design also limits epidemiologic interpretation. The present study was not designed as a population-based or cohort study and therefore does not provide a source-population denominator or a predefined control group. In addition, the available reports of hereditary β-thalassemia with MDS or related myeloid neoplasms are primarily case-based and do not provide a consistent denominator for pooled prevalence estimation.^[11–15]^ We therefore did not calculate a co-occurrence prevalence or perform a control-group analysis, because such estimates would not be methodologically supported by the available data. Future multicenter cohort or registry-based studies with systematic hemoglobinopathy assessment and defined comparator populations would be required to address this epidemiologic question.

The relatively short follow-up period, comprising only three outpatient visits over approximately three months, represents an important limitation of this report. Although the patient’s clinical condition and hematologic findings remained generally stable during the available observation period, this short-term stability should not be interpreted as evidence of durable disease stability or a favorable long-term prognosis. The available data were insufficient to assess subsequent transfusion dependence, clonal progression, leukemic transformation, or longer-term treatment requirements. Longer longitudinal follow-up would be required to characterize the clinical course of SF3B1-mutated MDS in the setting of hereditary β-thalassemia trait.

Overall, the diagnostic trajectory underscores the importance of integrating longitudinal hematologic history, red-cell indices, hemoglobin analysis, bone marrow morphology, and molecular findings when evaluating microcytic anemia in a patient with a clonal myeloid disorder.

## 4. Conclusion

In summary, this report describes SF3B1-mutated MDS in a patient with β-thalassemia trait, strongly supported by longitudinal hematologic findings, quantitative hemoglobin analysis, and HBB testing. The principal clinical value of this case is the distinction between a long-standing hereditary cause of microcytosis and an acquired thalassemic phenotype related to the myeloid clone. Longitudinal hematologic findings, hemoglobin analysis, bone marrow morphology, and molecular testing supported the coexistence of two distinct disorders. The available case-based literature is insufficient to determine the prevalence of this co-occurrence or to establish an epidemiologic association between β-thalassemia trait and MDS.^[11–15]^

## Acknowledgments

We thank Shunhong Zhuang for comments on the manuscript.The authors declare no conflicts of interest.

## Author contributions

**Conceptualization:** Zenghui Fang, Lu Chen.

**Data curation:** Zenghui Fang, Menghe Zhao.

**Formal analysis:** Zenghui Fang, Lu Chen.

**Investigation:** Zenghui Fang, Lu Chen, Menghe Zhao.

**Writing – original draft:** Zenghui Fang, Xin Chen.

**Writing – review & editing:** Zenghui Fang, Xin Chen.

**Funding acquisition:** Lu Chen, Xin Chen.

**Resources:** Lu Chen, Xin Chen.
